# The *caenorhabditis elegans *CDT-2 ubiquitin ligase is required for attenuation of EGFR signalling in vulva precursor cells

**DOI:** 10.1186/1471-213X-10-109

**Published:** 2010-10-26

**Authors:** Gino B Poulin, Julie Ahringer

**Affiliations:** 1 Faculty of Life Sciences, University of Manchester, Michael Smith Building, Oxford Road, Manchester, M13 9PT, UK; 2 The Gurdon Institute, University of Cambridge, Tennis Court Road, Cambridge, CB2 1KN, UK

## Abstract

**Background:**

Attenuation of the EGFR (Epidermal Growth Factor Receptor) signalling cascade is crucial to control cell fate during development. A candidate-based RNAi approach in *C. elegans *identified CDT-2 as an attenuator of LET-23 (EGFR) signalling. Human CDT2 is a component of the conserved CDT2/CUL4/DDB1 ubiquitin ligase complex that plays a critical role in DNA replication and G2/M checkpoint. Within this complex, CDT2 is responsible for substrate recognition. This ubiquitin ligase complex has been shown in various organisms, including *C. elegans*, to target the replication-licensing factor CDT1, and the CDK inhibitor p21. However, no previous link to EGFR signalling has been identified.

**Results:**

We have characterised CDT-2's role during vulva development and found that it is a novel attenuator of LET-23 signalling. CDT-2 acts redundantly with negative modulators of LET-23 signalling and CDT-2 or CUL-4 downregulation causes persistent expression of the *egl-17::cfp *transgene, a marker of LET-23 signalling during vulva development. In addition, we show that CDT-2 physically interacts with SEM-5 (GRB2), a known negative modulator of LET-23 signalling that directly binds LET-23, and provide genetic evidence consistent with CDT-2 functioning at or downstream of LET-23. Interestingly, both SEM-5 and CDT-2 were identified independently in a screen for genes involved in receptor-mediated endocytosis in oocytes, suggesting that attenuation of LET-23 by CDT-2 might be through regulation of endocytosis.

**Conclusions:**

In this study, we have shown that CDT-2 and CUL-4, members of the CUL-4/DDB-1/CDT-2 E3 ubiquitin ligase complex attenuate LET-23 signalling in vulval precursor cells. In future, it will be interesting to investigate the potential link to endocytosis and to determine whether other signalling pathways dependent on endocytosis, *e.g*. LIN-12 (Notch) could be regulated by this ubiquitin ligase complex. This work has uncovered a novel function for the CUL-4/DDB-1/CDT-2 E3 ligase that may be relevant for its mammalian oncogenic activity.

## Background

*C. elegans *vulva development has been instrumental in the characterisation of numerous major signalling pathways such as EGFR (Epidermal Growth Factor Receptor), and Notch [1,2]. Even though most of the components of these core signalling pathways have been identified, the modulatory mechanisms remain difficult to decipher because of the intricate network formed by negative and positive feedback loops. In an attempt to identify novel players in attenuation of LET-23 (EGFR) signalling, we used a candidate-based approach to screen, by RNAi, for genetic interactors of *gap-1 *(see materials and methods). Deletion of *gap-1 *creates a hypersensitive background for the LET-23(EGFR)/LET-60(RAS)/LIN-45(RAF) signalling cascade, in which the loss of an additional attenuator can cause the Multivulvae (Muv) phenotype [2-4]. This strategy identified CDT-2, an evolutionary conserved homologue of human CDT2 also called DTL or DCAF2.

Human CDT2 was first discovered as a transcript down regulated following retinoic acid-induced neuronal differentiation in pluripotent NT2 cells [5], suggesting a role in maintenance of self-renewal capacity. CDT2, a WD40 domain containing protein, was later found associated to the CUL4/DDB1 E3 ubiquitin ligase complex [6-8]. Within this complex, CDT2 acts as a substrate recognition subunit. Two substrates have been well characterised: the license to replicate, CDT1, and the CDK inhibitor, p21 [7,9-13]. Degradation of CDT1 and p21 are essential to prevent rereplication or firing of origin of replication following DNA damage-induced stress. Therefore, CDT2, as part of the CUL4/DDB1 E3 ubiquitin ligase complex, plays a critical role in regulation of DNA replication.

Mouse CDT2 activity is essential for viability, which has precluded the study of its role during development [14]. RNAi in *C. elegans *can sometime create knock down conditions that allow the identification of novel late onset activities. Here, we show that *C. elegans *CDT-2 and CUL-4 attenuate LET-23 signalling during vulva development. We found that SEM-5 (GRB2) physically interacts with CDT-2 and genetic studies are consistent with CDT-2 acting at the level of the LET-23 receptor. Finally, we confirmed that CDT-2 and SEM-5 are required for receptor-mediated endocytosis in oocytes [15]. We propose a model by which the CUL-4/DDB-1/CDT-2 ubiquitin ligase complex associates with SEM-5 to target LET-23 and regulate its endocytosis.

## Methods

### Strains and general maintenance

Strains were maintained as described in Brenner, [16]. *lin-15AB(n765) *is a temperature sensitive allele, which produces a lower penetrance Muv phenotype at 15°C than at 25°C. Strains genotypes are: *gap-1(n1691)*, *gap-1(ga133)*, *lin-3(n378)*, *let-23(sy1)*, *let-60(n1046)*, *lin-15A(n767)*, *lin-15B(n744)*, *dpy-23(e840)*, *sem-5(n1779)*, *sli-1(sy143)*, *unc-101(sy108)*, *cul-4(gk511)/mIn1[mIs14 dpy-10(e128)], unc-4(e120) II; arIs92[egl-17p::CFP::lacZ, unc-4 (+), ttx-3p::GFP], unc-119(ed3);pwIs23[vit-2::gfp] unc-119(+), pwIs116(rme- 2::GFP)*.

### RNAi procedure

Briefly, worms for RNAi exposure were synchronised using standard bleaching to isolate embryos. These were grown to the L3 stage and then transferred to RNAi plates. The mothers were transferred onto a new plate after three days and the F1 s laid on this plate were analysed, as previously described [17]. For *lin-3_rf_*, *let-23_rf_*, and *lin-45_rf _(*and doubles with *gap-1*), F2 s were analysed instead of F1 s since the Vul phenotype leads to small broods. RNAi clones used in this study were all confirmed by sequencing.

### Scoring of the Multivulvae (Muv) phenotype

**Figure 1:**
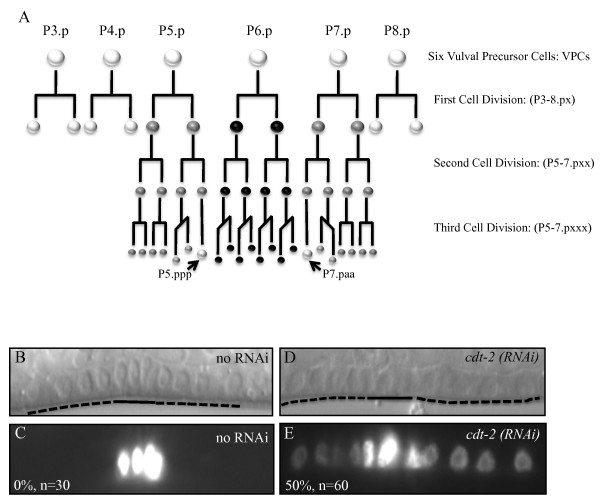
**Cell lineage of the vulva**. (A) Six Vulval Precursor Cells (VPCs) called P3.p to P8.p have the potential to adopt the vulval fate [47,48]. The anterior of the animal is on the left (P3.p) and posterior on the right (P8.p). In wild type animals only three VPCs (P5.p -P7.p) will adopt the vulval fate, the other VPCs will fuse to hyp7 after first division (white circles). Sometime, P3.p fuses before the first cell division. The VPCs that are committed towards the vulval fate will undergo three rounds of cell division. The descendants after first division are named P5/6/7.px, and after second division P5/6/7.pxx, and after third divison P5/6/7.pxxx (x signify either anterior cell (a) or posterior cell (p)). Of note, two cells will not undergo the third division (see arrows). The descendants of P6.p are classified as primary cells, and express high levels of LET-23 signalling (black circle). Descendants of P5.p and P6.p are classified as secondary and express low levels of LET-23 signalling (grey circles). The lines indicate the axis of cell division, which is longitudinal for all cells up to second division. However, at third division, all the primary cells will divide in the transverse axis of cell division, as well as two cells that are descendants of P5.p and P7.p. (B, C, D and E) Depletion of *cdt-2 *causes excessive LET-23 signalling in secondary cells. DIC and fluorescent photos showing that *egl-17::cfp *is only expressed in primary cells (underlined) and not in the secondary cells (broken line) of wild type animals (B and C). However, in animals depleted of *cdt-2*, secondary cells (broken line) express *egl-17::cfp*, indicating excessive LET-23 signalling in those cells (D and E). % of animals with persistent expression of *egl-17::cfp *is indicated at the bottom right with the number of worms analysed.

Induction of vulval cells was scored by lineage analysis of vulval precursor cells (VPCs). Briefly, L4 animals were mounted on agarose pads and the descendants of the six Vulval Precursors Cells (VPCs) analysed to assign either the vulval fate or the non-vulval fate (fusion to hyp 7) (Figure 1, assay done as in POULIN*et al*. 2005). Each fully induced VPC is given a score of 1; a wild-type vulva therefore has a score of three because three VPCs adopt the vulval fate. A score of 0.5 is given if only one VPC daughter produces vulval tissue and one fuses with hyp7. A score of 0 is given if the VPC or both of its daughters fuse with hyp7.

Statistical analysis was performed mostly using the Mann-Whitney U-test with the calculated number of VPCs induced, except for the *lin-3_rf _*analysis on which the Fisher's exact test was performed to analyse the proportionality of control worms with wild type vulva compared to *cdt-2(RNAi) *worms. In this particular case, it is likely that the Mann-Whitney test introduced a type II error (acceptance of the null hypothesis).

### *egl-17::cfp *assay

Briefly, L3 animals were mounted on agarose pads and examined for persistent expression of *egl-17::cfp *in secondary cells (light grey cells in Figure 1). These cells do not normally express *egl-17::cfp *at this stage (after second or third division) [18]. Importantly, this assay must be performed prior to L4, since *egl-17 *expression disappears from the primary cells and appears in secondary cells at mid-L4 [19]. For the analysis of the *cul-4 *mutants, heterozygous and homozygous animals were analysed in parallel, and were from the same mothers. Therefore the analysed animals were at roughly the same age in absolute time.

### vit-2::gfp assay

Briefly, the *vit-2::gfp *assay was performed as described [20], and RNAi perform as indicated above. Young adults were analysed and animals with gross gonadal defects were not analysed as they could bias the assay.

### *In vitro *pull down assay

Briefly, CDT-2 was produced using an *in vitro *transcription/translation reaction according to the manufacturer (Promega, Reticulocyte Lysate System, Non-Radioactive labelling). SEM-5 and SLI-1 were fused to GST and purified on column according to manufacturer. The pull down was performed as previously described [21]. Equivalent amount of GST fusion proteins (~200 ng) were used per pull down, the size of the proteins visualised on gels stained by Coomassie, and protein concentrations measured by Bradford assay.

### Microinjection for translational *cdt-2::gfp *transgenic

The *cdt-2::gfp *transgene (JA1501, *weEx70*[*cdt-2p::cdt-2::gfp::let-858(3'UTR) + pha-1(+)*] was generated by cloning DNA containing 3 kb upstream of the *cdt-2 *start codon and the entire *cdt-2 *coding region into plasmid pSB_GW::GFP containing GFP (as a C-terminal fusion) and the *let-858 *3'UTR. Transgenic animals were made by microinjection [22], selected by co-injecting *pha-1(e2123) *with the transgene and a plasmid containing the *pha-1 *gene [23]. Functionality of the transgene was not tested.

## Results

### *cdt-2 *genetically interacts with *gap-1*

We previously identified *cdt-2 *as having a potential role in vulva development because knockdown caused a weak synMuv (synthetic Multivulvae) phenotype [17]. RNAi caused a low penetrance synMuv phenotype in a *lin-15A *background, but it did not pass the penetrance threshold and therefore was not further analysed at the time. It was subsequently shown that *lin-15A *could act redundantly with *gap-1 *to prevent erroneous adoption of vulval fate [24]. GAP-1 is a GTPase Activating Protein that acts as an attenuator of LET-60 RAS signalling, and the *gap-1 *mutant has been shown to be a sensitized background for identifying attenuators [18]. The interaction with *lin-15A *suggested that some of the weak candidate synMuv genes we previously identified might genetically interact with *gap-1*.

We therefore tested whether *cdt-2 *could interact with *gap-1 *to cause a Muv phenotype. We found that *cdt-2(RNAi) *in the *gap-1 *background causes 43% of animals to present a Muv phenotype (Table 1). We also confirmed that *cdt-2 *only marginally interacts with *lin-15A *or *lin-15B *(Table 1). In addition, RNAi of *cdt-2 *slightly increases penetrance of the Muv phenotype observed in a *lin-15AB *mutant (Table 1), which is consistent with an atypical synMuv activity.

**Table 1:** *cdt-2 *interacts with *gap-1 *to produce the Muv (Multiple vulvae) phenotype

	*cdt-2(RNAi)*		no RNAi	
	
genotypes	Muv %	VPCs (N)	Muv %	VPCs (N)
N2 *gap-1(n1691)*	0 43	3.0 (30) 3.4 (76)	0 0	3.0 (many) 3.0 (30)
*lin-15A(n767)*	7.5	3.1 (40)	0	3.0 (many)
*lin-15B(n744)*	2	3.0 (55)	0	3.0 (many)
*lin-15AB(n765)*^a^	33	3.3 (30)	13	3.1 (30)

^a^Temperature sensitive allele maintained at 15°C

Animals are Muv if more than 3 VPCs (Vulval Precursor Cells) adopt the vulval fate

### CDT-2 prevents excessive LET-23 EGFR signalling during vulva development

The genetic interaction observed with *gap-1 *suggested that *cdt-2 *could be involved in attenuation of LET-23/LET-60/MPK-1 signalling. Therefore, we addressed whether depletion of *cdt-2 *could cause excessive LET-23/LET-60/MPK-1 signalling in a 'non-redundant' fashion as previously described for *gap-1*, other negative modulators of LET-60 (RAS) signalling [18,25], and a subset of synMuv genes [26]. To this end, we used *egl-17::cfp *(see materials and methods), a reporter for excessive LET-23/LET-60/MPK-1 signalling during vulva development [18]. In wild-type animals, *egl-17::cfp *is only expressed in primary cells at the third larval stage (Figure 1A, black circles, and 1B and C). However, under conditions of excess LET-23/LET-60/MPK-1 signalling, *egl-17::cfp *expression persists in secondary cells (Figure 1A, grey circles). We found that depletion of *cdt-2 *by RNAi causes persistent expression of *egl-17::cfp *in P5.p and P7.p descendant cells of 50% of the animals analysed (Figure 1D and 1E). Taken together, the genetic interaction with *gap-1 *and the persistent expression of *egl-17::cfp*, strongly suggest that CDT-2 is an attenuator of LET-23/LET-60/MPK-1 signalling during vulva development.

### CUL-4 prevents excessive LET-23 EGFR signalling during vulva development

Mammalian CDT2 has been found associated with the CUL4/DDB1 ubiquitin ligase complex [6-8], which prompted us to test whether the *C. elegans *homologues of the complex would possess an activity similar to CDT-2. RNAi of *cul-4*, *ddb-1*, or *rbx-1 *(also found in this complex [27,28]) did not produce a Muv phenotype in the *gap-1 *background (data not shown), but the rereplication phenotype [13,29] could be detected in these experiments (data not shown). Because RNAi knockdown animals might retain residual activity, we also investigated the phenotype of a *cul-4 *deletion mutant.

**Figure 2:**
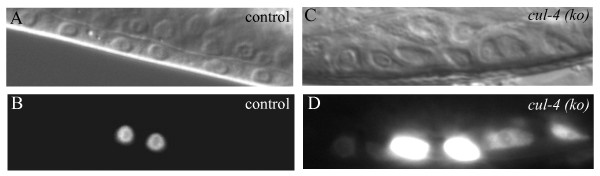
**Persistent expression of *egl-17::cfp *in *cul-4 *knock out worms**. (A and B) A control animal that shows the expected expression of *egl-17::cfp *in the primary cells after first division. (C and D) A *cul-4 *knock out *(ko) *animal in which *egl-17::cfp *expression is observed in the flanking secondary cells also after first division.

Using a *cul-4 *knock out strain and the *egl-17::cfp *assay, we assessed a possible role of *cul-4 *in attenuation of LET-23 signalling. Although *cul-4 *homozygotes arrest development as larvae and do not complete vulva development [13,29], the vulval precursor cells can undergo one cell division, allowing assay of persistent *egl-17::cfp *expression in secondary P(5/7).px cells (Figure 1A). We found that *egl-17::cfp *expression persists in secondary cells after first division (Table 2 and Figure 2). At this stage, 75% of the *cul-4 *homozygotes (*cul-4/cul-4) *had persistent expression compared to 10% of heterozygotes (*cul-4/+)*. We obtained similar results analysing P(5/7).p cells: 62.5% of *cul-4/cul-4 *animals have persistent expression of *egl-17::cfp *compared to 18% of *cul-4/+ *animals (Table 2). These results suggest that *cul-4*, similar to *cdt-2*, has a role in preventing excess LET-23/LET-60/MPK-1 signalling during vulva development.

**Table 2:** Deletion of *cul-4 *causes persistent expression of *egl-17::cfp*

	One-cell stage		Two-cell stage	
	
genotypes	**% Persistent exp**.	N	**% Persistent exp**.	N
*cul-4/cul-4*	62.5	24	75	8
*cul-4/+*	18	22	10	20
N2	6	18	0	30

### CDT-2 is expressed in dividing vulval precursor cells

**Figure 3:**
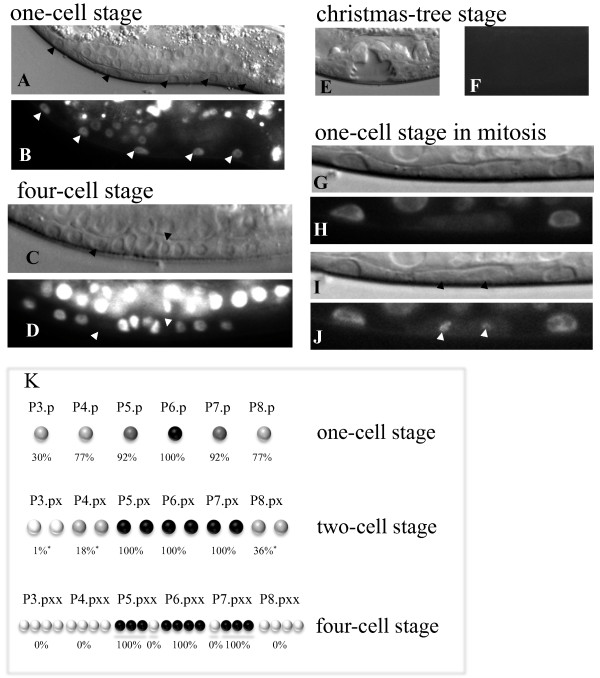
**Pattern of expression of CDT-2::GFP during vulva development**. P3.p-P8.p cells express CDT-2 (A and B), but sometimes (30%) P3.p does not (K), perhaps reflecting fusion to hyp7 prior to first cell division (see Figure 1). At four-cell stage, two cells that will not proceed to the third division stop expressing CDT-2 (C, D and K). At Christmas-tree stage CDT-2 is not detected (E and F). Sequential pictures of P6.p undergoing first division showing that during mitosis CDT-2 can be detected in cytoplasm (G and H), and later that CDT-2 is relocalised at the nuclear membrane (I and J). Frequency of expression observed in vulval cells at different stages (K). Black or dark grey circles represent high frequency of expression (100% and 92%). Light grey circles represent a low to medium frequency of expression (18, 30, 36 or 77%). White circles represent undetected or very low frequency of expression (0 or 1%).

Since CDT-2 plays an important role during vulva development, we analysed its expression using a translational GFP fusion. The fusion protein is predominantly nuclear, as has been seen for other CDT2 homologs. CDT-2::GFP is not detected in P cells at larval stage L1 (data not shown), but is expressed early in all Vulval Precursor Cells (VPCs, Pn.p cells) prior to their first division (Figure 3A and 3B). The frequency of expression is lowest in P3.p cells, and highest in P6.p (Figure 3K). After first division, the cells that adopted the vulval fate all express CDT-2::GFP, but the non-vulval cells generally do not (Figure 3K, two-cell stage). However, sometimes low expression can be observed in the descendants of P3.p, P4.p and P8.p (Figure 3K, two-cell stage). Interestingly, after second division (four-cell stage) CDT-2::GFP expression disappears from two secondary cells (P5.ppp and P7.paa, see Figure 1); these are the only vulval cells that will not undergo a third cell division (Figure 3C, D and 3K). Later, at L4 stage no expression is detected (Figure 3E and 3F). We also observed CDT-2:GFP expression in the cytoplasm during the first mitotic division of P6.p, which quickly relocalised to the nuclei as the nuclear envelope reforms (Figure 3G-I). The early CDT-2 pattern of expression is consistent with a role during vulval fate adoption, and its down regulation in cells that cease cell division is consistent with a role in DNA replication.

### CDT-2 is active at the level of the LET-23 receptor and physically interacts with SEM-5

To try to understand how CDT-2 attenuates the LET-23 signalling cascade during vulva development, we analysed the type of epistatic interactions produced between *cdt-2(RNAi) *and reduced function (rf) alleles of *lin-3/Egf*, *let-23/Egfr*, and *lin-45/Raf*. We first tested whether depletion of *cdt-2 *could rescue the Vul (vulvaless) phenotype produced by *lin-3_rf_*, *let-23_rf_*, or *lin-45_rf_*. Depletion of *cdt-2 *by RNAi did not affect the penetrance of the Vul phenotype produced by *lin-45_rf_*, but did partially suppress the Vul phenotype of *let-23_rf _*(Table 3). RNAi of *cdt-2 *in *lin-3_rf _*also affected the penetrance of the Vul phenotype (2% of control animals with a wild type vulva *versus *19% of *cdt-2(RNAi) *animals, (Fisher's test, p = 0.002)) (Table 3), indicating that the Vul phenotype caused by a reduction of ligand can be rescued. Of note, the *lin-3_n378 _*allele used here is a reduced function allele that was shown to still retain ligand activity [30]. We obtained similar results performing epistasis experiments in a sensitized *gap-1 *mutant background (Table 3). Depletion of *cdt-2 *did not rescue the Vul phenotype of the *lin-45_rf_;gap-1 *double but did increase the penetrance of the Muv phenotype of *let-23_rf_;gap-1 *double mutants, as well as the number of VPCs induced (Table 3). A similar trend was seen with *lin-3_rf_;gap-1*, though not statistically significant (Table 3). Depletion of *cdt-2 *also enhanced the penetrance of the Muv phenotype and the number of VPCs induced in a *let-60 *gain of function allele (Table 3). Taken together, these results are consistent with *cdt-2 *acting upstream of *lin-45*, but downstream or at the level of *let-23 *to attenuate this signalling cascade.

**Table 3:** Epistasis between *cdt-2 *and components of the EGF signalling cascade

	*(RNAi) cdt-2*			no RNAi		
	
genotypes	Muv %	Vul %	VPCs (N)	Muv %	Vul %	VPCs (N)
*lin-3(n378)*	0	81	0.83 (75)	0	98	0.49 (75)
let-23(sy1)**	7	58	1.97 (46)	0	87	0.72 (30)
let-60(n1046)***	96	0	4.86 (24)	66	0	3.70 (30)
*lin-45(n2018)*	0	28	2.43 (36)	0	17	2.68 (30)
*lin-3(n378);gap-1(n1691)*	0	3	2.97 (60)	0	10	2.77 (60)
let-23(sy1);gap-1(ga133)***	90	0	4.50 (50)	70	0	3.80 (50)
*lin-45(n2018);gap-1(n1691)*	0	10	2.88 (30)	0	7	2.95 (30)

***P < 0.001, **P < 0.01

We further analysed the capacity of *cdt-2 *to genetically interact with other negative modulators of the LET-23 signalling pathway that are known to act at the level of the receptor. Previous work showed that UNC-101 and DPY-23 are adaptins orthologous to the mu1 and mu2 subunits of adaptor protein complex 1 and 2 (AP1 and AP2, respectively), and that they both can act as negative modulators of LET-23 signalling [18,31]. Similarly, SLI-1 is orthologous to CBL, an E3 ubiquitin ligase targeting LET-23 for degradation [4,32] and SEM-5 is GRB2, an adaptor molecule that physically interact with EGFR [2,33,34]. To address whether these genes could interact with *cdt-2*, we used loss of function (lf) alleles of *dpy-23/AP2*, *unc-101/AP1*, *sli-1/CBL*, and *sem-5/GRB2 *and performed *cdt-2(RNAi)*. We found that *cdt-2 *genetically interacts with *dpy-23_lf _*and *unc-101_lf_*, as *cdt-2 *RNAi induces a Muv phenotype in these backgrounds. In contrast, no interaction was seen with *sli-1_lf _*or *sem-5_lf _*(Table 4).

**Table 4:** Epistasis between *cdt-2 *and negative modulators of EGF signalling

	*(RNAi) cdt-2*		no RNAi	
	
genotypes	Muv %	VPCs, (N)	Muv %	VPCs, (N)
*dpy-23(e840)*	36	3.27, (22)	0	3.0, (27)
*sem-5(n1779)*	0	2.71, (28)	0	2.83, (18)
*sli-1(sy143)*	0	3.0, (90)	0	3.0, (30)
*unc-101(sy108)*	20	3.2, (30)	2	3.01, (45)

**Figure 4:**
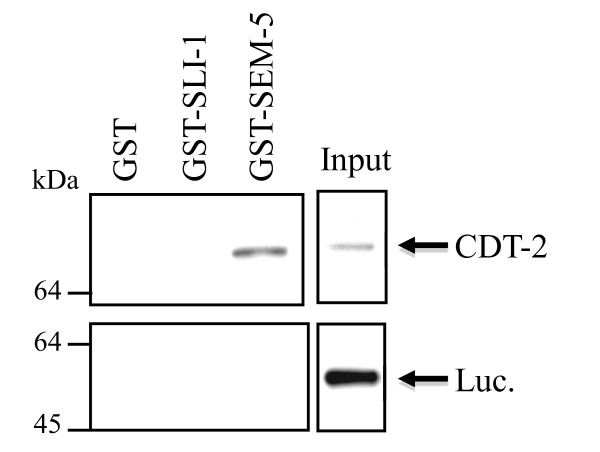
**CDT-2 can interact physically with SEM-5, the *C. elegans *GRB2 homologue**. Pull down using *in vitro *labelled CDT-2 and purified GST, GST::SLI-1 or GST::SEM-5 shows that SEM-5 but not SLI-1 or GST can interact with CDT-2. As an additional negative control, we used labelled luciferase and did not detect interactions with either SEM-5 or SLI-1. Input is set at 5%.

Since an absence of genetic interaction can sometimes suggest a physical interaction [35,36], we tested whether CDT-2 could physically interact with either SLI-1 or SEM-5. We produced *in vitro *labelled CDT-2 and purified SLI-1 and SEM-5 from bacteria. We found that CDT-2 could physically associate with SEM-5, but not with SLI-1 (Figure 4). Together, the genetic and physical interaction data suggest that CDT-2 may prevent excessive signalling regulating LET-23 through SEM-5.

### Depletion of CDT-2 or SEM-5 causes similar receptor-mediated endocytosis defect

**Figure 5:**
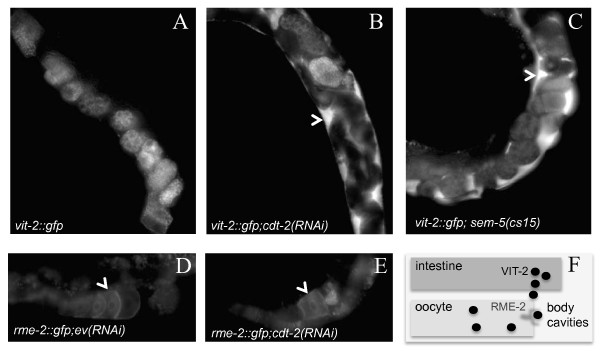
**Depletion of *cdt-2 *causes a similar endocytotic defect than observed in a loss of function allele of *sem-5***. We analysed the uptake of VIT-2, yolk, using a *vit-2::gfp *expressing strain (A) and observed accumulation in body cavities in *cdt-2(RNAi) *(B) and in *sem-5 *loss of function (C). Arrows point to sites that accumulated VIT-2::GFP. We verified that the receptor, RME-2, is properly expressed using an *rme-2::gfp *expressing strain. No difference in oocytes expressing RME-2 could be observed when the control (*empty vector (RNAi)*) was compared to *cdt-2(RNAi) *animals (D and E). Arrows indicate oocytes.

The association between CDT-2 and SEM-5 suggests that they function together in a common process. Interestingly, both *sem-5 *and *cdt-2 *have been identified in an RNAi screen designed to identify genes required for receptor-mediated endocytosis in oocytes [15]. The assay used in this screen is based on the accumulation of VIT-2::GFP (yolk) in body cavities. VIT-2 is secreted into the body cavities by the intestine and is endocytosed by oocytes *via *the yolk receptor, RME-2 ([20] and Figure 5F). By fusing VIT-2 to GFP, it is possible to assess whether receptor-mediated endocytosis is functional, because if not VIT-2::GFP accumulates in body cavities of young hermaphrodites [20]. We confirmed that reduction of *cdt-2 *or *sem-5 *causes body cavity accumulation of the *vit-2::gfp *reporter (Figure 5A-C).

Because correct cortical localization of the RME-2 yolk receptor is required for endocytosis [20], we next examined receptor localization in *cdt-2 *RNAi animals to test whether the accumulation of *vit-2::gfp *might be caused indirectly by improper localization of the receptor. We found that the expression and localization of an *rme-2::gfp *reporter is normal in *cdt-2(RNAi) *animals (Figure 5D and 5E). The correct localization of RME-2::GFP combined with the defect in uptake of VIT-2::GFP suggests that CDT-2 plays a role (direct or indirect) in the process of receptor-mediated endocytosis.

## Discussion

CDT2 is a recognition subunit of the CUL4/DDB1 E3 ubiquitin ligase complex important for DNA replication and G2/M checkpoint [9,10,27,28]. Previous work has shown that these functions are conserved in *C. elegans *[11-13,29]. We have uncovered a novel role for CUL-4 and CDT-2 in preventing excess LET-23 signalling. Because CDT-2 and CUL-4 normally work as part of the CDT-2/CUL-4/DDB-1 ubiquitin ligase complex, it seems likely that DDB-1 also participates in attenuation of LET-23 signalling. However, RNAi of *ddb-1 *did not cause obvious LET-23 signalling defects. This might be due to incomplete knockdown, or alternatively, CDT-2 and CUL-4 could act independently of DDB-1 in this context. We also provided *in vitro *evidence that CDT-2 can associate with SEM-5 directly. CDT-2 and SEM-5 share two functions: they attenuate LET-23 signalling during vulva development and are required for receptor-mediated endocytosis during oogenesis. Linking these two functions together, we hypothesise that the CUL-4/DDB-1/CDT-2 E3 ubiquitin ligase might interact with SEM-5 to affect LET-23 endocytosis and attenuation of the LET-23 signalling cascade. However, our studies do not rule out an effect through other signalling pathways involved in vulva development such as Wnt or Notch (see below).

### The rereplication defect and LET-23 signalling

The rereplication defect caused by depletion of CDT-2 or CUL-4 has been previously characterised as well as the cell cycle arrest phenotype [11,13,29]. However, it is difficult to explain that these defects could cause excessive LET-23 signalling during vulva development. Indeed, experiments using hydroxyurea to arrest the VPC cell cycle have shown that *egl-17 *expression remains restricted to P6.p [37]. Therefore, a replication block after first division as in the case of *cul-4 *deletion mutants is unlikely to cause persistent expression of *egl-17::cfp*. Furthermore, we observed increased LET-23 signalling in *cdt-2 *RNAi animals, and an increase in vulval fate adoption in *gap-1; cdt-2(RNAi) *animals, under conditions where the cell cycle proceeds normally. Therefore, the role of CDT-2 in preventing rereplication is likely to be independent of its function in preventing excess LET-23 signalling.

### CDT-2 may attenuate LET-23 signalling as a component of the CUL-4/DDB-1 E3 ligase complex

RNAi by feeding in *C. elegans *has significant false negative rate, but false positives are rare [38]. Hence, the finding that a deletion of *cul-4 *can cause the same vulval phenotype (*egl-17::cfp *assay, Table 2) as *cdt-2(RNAi) *suggests that both CUL-4 and CDT-2 are novel attenuators of LET-23 signalling. Since purification of the human CUL-4/DDB-1 E3 ligase complex by different groups has identified CDT-2 as the substrate recognition unit, it is likely that CUL-4 and CDT-2 function together in the process of LET-23 attenuation. Even though, this study cannot rule out that CUL-4 could act in parallel to attenuate LET-23 signalling.

### SEM-5 and attenuation of signalling

SEM-5, the GRB2 homologue, has two activities linked to Receptor Tyrosine Kinase (RTK) signalling. It can act as a positive regulator of signalling by recruiting SOS-1 [2,33,34], or act as a negative modulator by recruiting SLI-1, the CBL homologue [4,32]. SLI-1 is an E3 ubiquitin ligase that can associate with SEM-5 to target RTKs and promote lysosomal degradation [39]. Here we show that CDT-2 can physically interact with SEM-5 and genetic analyses are consistent with action at the level of the LET-23 receptor. This suggests that SEM-5 might regulate attenuation of LET-23 signalling through ubiquitination and subsequent endocytosis using two different ubiquitin ligases: SLI-1 (CBL) and CUL-4/DDB-1/CDT-2. Unfortunately, we have been as yet unable to directly assess LET-23 receptor localisation or endocytosis during vulva development; immunostaining experiments are inconsistent and current *let-23::gfp *transgenics are not fully functional. Tests of these models will require better reagents to investigate regulation of the LET-23 receptor.

### Ubiquitination and regulation of Notch signalling

Receptor-mediated endocytosis is important to terminate or attenuate signalling [40], not only for EGFR but also for other signalling pathways, *e.g*. Notch. During vulva development, LIN-12 (Notch) signalling is required for establishment of the secondary cell fate [41] and for the production of the anchor cell, which produces LIN-3 (EGF) [42]. Interestingly, SEL-10, an F-box and a WD40 containing protein that belongs to the CDC4/CUL-1 family of ubiquitin ligase [43], has been shown to play an important role in attenuation of LIN-12 signalling [44]. SEL-10 was also shown to physically interact with LIN-12, implying that it regulates signalling *via *ubiquitination of LIN-12. Herein we have not investigated the relationship between the CUL-4/DDB-1/CDT-2 ubiquitin ligase complex and LIN-12 signalling. We did not observe any defects in anchor cell development (unpublished data), a process dependent on LIN-12, however, it has been previously shown for SEL-10 that a sensitised background is required to reveal its activity as an attenuator of LIN-12 signalling [44]. Therefore, we may not have detected a potential role for CDT-2 in attenuation of LIN-12 signalling.

There is also an intimate link between LIN-12 and LET-23 signalling during vulva development [44]. Indeed, high level of LET-23 signalling triggers expression of LIN-12 ligands in the primary P6.p cell [45]. This activates LIN-12 signalling in the flanking secondary cells and ensures down regulation of LET-23 signalling in P5.p and P7.p cells [18,25,46]. It is not impossible that the depletion of CDT-2 or CUL-4 impairs LIN-12 signalling and thereby prevents appropriate down regulation of LET-23 signalling in secondary cells, which would cause persistent expression of *egl-17::cfp *in secondary cells.

### Localisation of CDT-2

The localisation of CDT-2 fused to GFP is predominantly nuclear in interphase and cytoplasmic during mitosis, which seems contrary with a function in endocytosis. However, we cannot exclude that a proportion of CDT-2::GFP below our limit of detection is cytoplasmic during interphase. Interestingly, early studies showed that human CDT-2 can be detected in the cytoplasm [5], which would be consistent with a role in ubiquitination of cytoplasmic targets. Alternatively, the CUL-4/DDB-1/CDT-2 E3 ubiquitin ligase complex may be active in the cytoplasm only after nuclear breakdown. Further experiments will be required to establish when and whether the complex is active in the cytoplasm, in particular during mitosis.

## Conclusion

We have identified CDT-2 and CUL-4 as novel attenuators of LET-23 signalling during vulva development. Both of these proteins are known components of a conserved CUL-4/DDB-1/CDT-2 E3 ubiquitin ligase complex, suggesting a novel function for this complex in signalling during *C. elegans *development. A similar function in other organisms might have been missed because of its requirement for cell cycle progression. Studying this complex in mammalian cells using knock down conditions that bypass the cell cycle defect might reveal a conserved role in signalling.

## Authors' contributions

GP performed and conceived all the experiments. JA contributed to experimental design and writing of the manuscript. Both authors read and approved the final manuscript.
